# Six shades lighter: a bit-serial implementation of the AES family

**DOI:** 10.1007/s13389-021-00265-8

**Published:** 2021-06-01

**Authors:** Sergio Roldán Lombardía, Fatih Balli, Subhadeep Banik

**Affiliations:** 1 Ecole polytechnique fédérale de Lausanne, Lausanne, Switzerland; 2 Security and Cryptography Laboratory (LASEC), Ecole polytechnique fédérale de Lausanne, Lausanne, Switzerland

**Keywords:** AES-128/192/256, Lightweight cryptography, Bit-serial implementation, Symmetric cryptography

## Abstract

Recently, cryptographic literature has seen new block cipher designs such as PRESENT, GIFT or SKINNY that aim to be more lightweight than the current standard, i.e., AES. Even though AES family of block ciphers were designed two decades ago, they still remain as the de facto encryption standard, with AES-128 being the most widely deployed variant. In this work, we revisit the combined one-in-all implementation of the AES family, namely both encryption and decryption of each AES-128/192/256 as a single ASIC circuit. A preliminary version appeared in Africacrypt 2019 by Balli and Banik, where the authors design a byte-serial circuit with such functionality. We improve on their work by reducing the size of the compact circuit to 2268 GE through 1-bit-serial implementation, which achieves 38% reduction in area. We also report stand-alone bit-serial versions of the circuit, targeting only a subset of modes and versions, e.g., AES-192 and AES-256. Our results imply that, in terms of area, AES-192 and AES-256 can easily compete with the larger members of recently designed SKINNY family, e.g., SKINNY-128-256, SKINNY-128-384. Thus, our implementations can be used interchangeably inside authenticated encryption candidates such as SKINNY-AEAD/-HASH, ForkAE or Romulus in place of SKINNY.

## Introduction

Lightweight cryptography has become in the past years a popular research area with new lightweight block ciphers like PRESENT [1], SKINNY [2] or GIFT [3] being proposed and studied, primarily with the objective to achieve small implementations in silicon to work in devices with limited space. However, AES is still undoubtedly the most widely used encryption algorithm worldwide, partly due to the fact that its security claims resisted two decades of cryptanalysis.

Many lightweight implementations target area minimization through various optimizations and a reduction of the data path to obtain small circuits with respect to the gate-equivalent (GE) metric. This is the approach followed by Jean et al. [4] that proposes an encryption/decryption circuit for AES-128 with less than 1600 GE using a 1-bit data path. These implementations are fit for applications which heavily prioritize area minimization over latency, and it is natural to expect that the resulting circuit requires much more than 8 times the clock cycles required by byte-serial implementations to perform an AES encryption/decryption. These include wearable devices, biometric implants, RFID devices which have tight space constraints but can make do with low communication bandwidth.

In most real-world applications, AES-128 is the go-to member of this family. However, with the possible advent of quantum computers, there is a tendency to move to larger key sizes, as the claimed security level is challenged by the Grover’s algorithm in the post-quantum setting. If we take NIST Post-Quantum Standardization as an example, out of 17 second round post-quantum KEM candidate constructions, 9 candidates use AES in their scheme. Eight of these candidates prefer AES-256 in counter mode, making it the clear contender for generating pseudo-randomness.

This encourages the research of lightweight implementation of the longer key siblings of AES family: AES-192 and AES-256. The work of Balli et al. [5] addresses these concerns by proposing a combined circuit, including encryption and decryption of the complete AES family using an 8-bit data path. It also addresses one common structure design challenge implementing both pipelines in a column-major fashion, as the standard explicitly recommends [6], in contrast to the row-major ordering preferred by some of the previous implementations [4, 7]. Deviating from the standard and assuming an ad hoc bit ordering always comes with a price, in the form of latency and area overhead to the surrounding circuit (i.e., the mode of operation that employs a block cipher), thus we avoid deviating from the standard.

### Previous work

There are already quite some number of works in the literature whose goal is to reduce the area cost of AES-128 (either encryption only or combined) as ASIC circuit. Satoh et al. propose a 32-bit-serial architecture with optimized tower field implementation of the S-box and a combinatorial optimization of the MixColumns circuit [8]. The size of this implementation is around 5400 GE (gate equivalents, i.e., occupied by an equivalent number of 2-input NAND gates). The *grain of sand* implementation [9] by Feldhofer et al. constructs an 8-bit serialized architecture with circuit size of around 3400 GE but a latency of over 1000 cycles for both encryption and decryption. The implementation by Moradi et al. [10] with size equal to 2400 GE and encryption latency of 226 cycles is one of the smallest known architectures for AES-128. This architecture is later improved by Banik et al. [11] such that the combined encryption and decryption circuit costs 2060 GE. In [12], the authors report an 8-bit-serial implementation that takes 1947/2090 GE for the encryption/decryption circuits, respectively. This implementation makes use of intermediate register files that can be synthesized in the ASIC flow using memory compilers, instead of classical flip-flops. Jean et al. proposed an implementation of AES-128 in a bit-serial way, focusing on area minimization and obtaining the smallest possible circuit known for this standard [4]. Their work achieves even further GE optimizations at the cost of latency.

More recently, Balli and Banik [5] proposed a combined implementation of AES-128/192/256 with an 8-bit path focusing on addressing security issues related to small keys in a post-quantum era. This work considers the aforementioned criteria and extends the results from the previous work for a combined circuit for AES-128/192/256 in a bit-serial fashion.

### Motivations

One of the main motivations, besides post-quantum trend, to build the smallest all-in-one AES in hardware is that some devices are expected to support large number of standards at the same time. For instance, many smart cards are designed to support a large variety of both symmetric and asymmetric cryptographic primitives, including all six functionalities of AES^1^. However, the number of protocols that these units can support is limited due to the tight area budget. Our design proposes an alternative combined solution with little extra area requirement, which would allow these cryptographic units to be able to benefit from the use of the full AES family without sacrificing significantly additional silicon budget. Besides, a combined implementation provides an upper bound on individual implementations of AES-192 and AES-256 that have not received sufficient attention in the literature.

Another major motivation to develop the combined circuit is the fact that many newer NIST post-quantum designs use AES-256 as a sub-primitive in randomness generation [13, 14]. Therefore, it is necessary to have constrained implementations of AES-256 in hardware without drastically increasing the area budget.

### Challenges

In our work, the main goal is to combine three versions AES-128, AES-192, AES-256 into single circuit in 1-bit-serial fashion. This essentially requires us to build a key pipeline that can flexibly accommodate variable length key (128, 192 or 256 bits), but still provide 128-bit round key at each round, similar to [5].

The first challenge we tackle is how to complete a round operation in 128 clock cycles, i.e., with the minimum latency possible in 1-bit serial setting. While this paper prioritizes the area minimization of the circuit, it does not overlook the latency. For encryption, byte-serial implementations complete a round in 21-24 clock cycles on average [5, 10, 11], whereas the previous work *bit-sliding* completes it in 168 clock cycles [4] (see Table 1 for comparison). In our design, we find a way to schedule AES state operations so that a round can be completed in 128 clock cycles, where both state and key pipelines operate in a non-stop fashion.

A second challenge is to produce 128 fresh bits of round key in 128 clock cycles. All-in-one AES circuit requires a key scheduling pipeline that can accommodate varying sizes of keys.

A previous work by Banik et al. [11] handles the key scheduling by interrupting large portions of the key pipeline (by using *clock-gating* to freeze flip-flops) during predetermined cycles. This approach is taken in order to efficiently share some circuit components between two pipelines, namely S-box. In order to avoid interruptions, we needed to carefully interweave the scheduling of S-box use between the state and the key pipelines.

Another challenge is that with longer keys, i.e., 192 and 256 bits, the round function and the key update operations are not synchronized, because each key update generates 192 (resp. 256) bits of key, whereas each round consumes exactly 128 bits. In particular, AES-192 only requires 8 full key update operations to produce enough key material for 12 rounds. Similarly, AES-256 only requires 7 full key updates to provide sufficient number of key bits for 14 rounds. Clearly, the synchronization then is no longer 1 round function call per key update, but 3 round function calls per 2 key updates for AES-192 and 2 round function calls per key update for AES-256. It should also be noted that the key update operation itself also varies based on the key length. The non-synchronization of AES-192 is especially challenging during decryption, which will be further explained in Sect. 5.3.

### Organization and contribution

**Table 1:** Comparison with the recent work

AES version	Serial	Area	Latency	References
		(GE)	(per round)	
128 Enc/Dec	32-bit	5400	5	[8]
128 Enc/Dec	8-bit	2060	23/31	[11]
128 Enc	8-bit	2400	21	[10]
128-192-256 Enc/Dec	8-bit	3672	24/32$^a$	[5]
128 Enc/Dec	1-bit	1596	168/248	[4]
128-192-256 Enc/Dec	1-bit	2268	128/128	this paper
192 Enc/Dec	1-bit	1906	128/128	this paper
256 Enc/Dec	1-bit	2004	128/128	this paper

Note that the area measures are taken for the smallest reported area results from each paper. $^a$[5] has different number of cycles for each round, and hence, the reported figures are computed as average

In the following section, we present a bit-serial architecture that performs AES-128/192/256 encryption and decryption and produce a circuit that can perform the 6 different functionalities. The circuit complies with the standard ordering of bits and avoids clock-gating technique. Both encryption and decryption operations take 1408, 1664 and 1920 clock cycles for AES-128, AES-192 and AES-256, respectively. The circuit occupies 2268 GE of area in silicon when synthesized with the standard cell library STM 90-nm CMOS logic process, which achieves an area reduction of 38% compared to the previous work *6-shades* [5] (under the same technology library).

The organization of the paper is as follows: Sect. 2 reminds AES internals. Section 3 presents the circuit components and primitives. Section 4 explains the data path circuit description and functionality in full details. Section 5 explains the key path in detail, and finally, the paper is concluded in Sect. 6 with reported area measurements.

## Background

In this section, we briefly revisit the AES standard. Namely, these are the state update and key expansion algorithms. It is assumed that the reader is familiar with AES. For more complete and detailed information, we refer the reader to the FIPS publication AES [6].

### Notation and AES overview

AES [6] defines a family of block cipher algorithms capable of encrypting and decrypting blocks of 128 bits using cryptographic keys of 128, 192 and 256 bits. AES, thus, specifies six functionalities, or *shades*, as each direction (i.e., either encryption or decryption) has fundamentally different operations at the circuit level. This variation requires us to design two complementary cores for each shade and combine them in a modular fashion. Namely, the data pipeline is dependent on the direction of operation, and the key pipeline is dependent on both the key length and the direction of operation at the same time.

Depending on the AES variant, let $$r $$ denote the number of rounds, $$l $$ denote the number of key derivation rounds, and $$b $$ denote the number of bytes of the initial key. Thus, each AES variant (or member) is associated with a tuple $$(r , l , b )$$ with values (10, 10, 16), (12, 8, 24) and (14, 7, 32) for AES-128, AES-192 and AES-256, respectively. We use $$d_0, d_1, \dots , d_{127}$$ to denote bits in the state values (or data), which is initialized either from plaintext or ciphertext. Equivalently, $$4 \times 4$$ state matrix *St* is also used to simplify some explanations in the text. Similarly, $$k_0, k_1, \dots , k_{x-1}$$ for $$x \in \{128, 192, 256\}$$ denotes the key. For a bit string $$d_0, d_1, \dots , d_{\ell }$$, we use $$d_{0:\ell }$$ as shorthand. We also use $$d_{x:y}$$ to denote its substring $$d_x, d_{x+1}, \dots , d_y$$ for some $$x<y$$.

We further assign variables to 1-bit storage elements of the circuit; namely, $\mathsf {FF}_x$ refers to the flip-flop identified with number *x*. Previous sequence notation is similarly extended, e.g., $\mathsf {FF}_{x:y}$ denotes the sequence of flip-flops $\mathsf {FF}_x, \mathsf {FF}_{x+1}, \dots , \mathsf {FF}_{y}$.

#### AES round function

At initialization, the plaintext $$d_{0:127}$$ (resp. the key) is encoded into $$4\times 4$$ state matrix $$St $$ in a column-first fashion [6], where each entry is a byte:

$$\begin{aligned}&\begin{bmatrix} St_{0,0},~ &{} St_{0,1},~ &{} St_{0,2},~ &{} St_{0,3} \\ St_{1,0},~ &{} St_{1,1},~ &{} St_{1,2},~ &{} St_{1,3} \\ St_{2,0},~ &{} St_{2,1},~ &{} St_{2,2},~ &{} St_{2,3} \\ St_{3,0},~ &{} St_{3,1},~ &{} St_{3,2},~ &{} St_{3,3} \end{bmatrix}\\&\quad = \begin{bmatrix} d_{0:7},~ &{} d_{32:39},~ &{} d_{64:71},~ &{} d_{96:103} \\ d_{8:15},~ &{} d_{40:47},~ &{} d_{72:79},~ &{} d_{104:111} \\ d_{16:23},~ &{} d_{48:55},~ &{} d_{80:87},~ &{} d_{112:119} \\ d_{24:31},~ &{} d_{56:63},~ &{} d_{88:95},~ &{} d_{120:127} \end{bmatrix} \end{aligned}$$

At each round, a series of operations is applied to the state *St* in the following order: SubBytes, ShiftRows, MixColumns and AddRoundKey. Before the first round, an additional AddRoundKey is executed to initialize the state using the plaintext and the initial key as inputs, and the last round skips the MixColumns call.

SubBytes substitutes each byte, according to the Rijndael S-box [15]. ShiftRows byte-wise rotates the *i*-th row by *i* to the left, for $0 \le i \le 3$. MixColumns multiplies each column with a predefined matrix *M* in the finite field $\mathsf {GF}(2^8)$. Finally, AddRoundKey returns the bit-wise XOR of the state and the corresponding round key.

#### AES key expansion

In order to obtain sufficiently long fresh key material for multiple calls of AddRoundKey operation, AES derives 128 bits of round key for each round by expanding the original encryption/decryption key. We recall and emphasize that for AES-192 and AES-256, the encryption/decryption keys are actually larger than 128 bits, and hence, each invocation of key expansion algorithm produces 192, 256 bits of round keys, respectively. This means that for AES-192, 2 key expansion calls are made for every 3 state rounds, and for AES-256, 1 key expansion call is made for every 2 state rounds.

Let $S :\{0,1\}^8 \rightarrow \{0,1\}^8$ denote the Rijndael S-box operation and the sequence $\mathsf {RC}_1, \dots , \mathsf {RC}_{10} \in \{0,1\}^8$ be the round constant bytes, as defined in the specification [6]. We abuse the key notation, and let $k_0, \dots , k_\ell$ denote the sequence of round key bits derived by scheduling an encryption key $k_0, \dots , k_{8r-1}$ for a particular choice of AES-128, AES-192 or AES-256 (where the initial bits of this sequence conveniently overlap with the key itself). Here, the length of the sequence is limited to $\ell \in \{1407, 1663, 1919 \}$, respectively, as these quantities define the total number of key bits used throughout encryption. Below, we briefly remind the key scheduling algorithm.

The key expansion call is made for 10, 8, 7 times for each version of AES-128, AES-192, AES-256, respectively. These number of calls generate sufficient number of bits because each state update consumes exactly 128 bits of round key, regardless of the key length of the AES version.

In particular, suppose that $k_{0:127}$ denotes the encryption key for $\textsf {AES-128}$. Let $i \in \{128, 256, \dots , 1280\}$. Then, the full sequence of key bits $k_{0:1407}$ is defined through the iteration of the key expansion algorithm. The subsequences $k_{i:i+31}$ are computed by:

$$\begin{aligned}&\begin{bmatrix} k_{i:i+7} \\ k_{i+8:i+15} \\ k_{i+16:i+23} \\ k_{i+24:i+31} \end{bmatrix} \leftarrow \begin{bmatrix} k_{i-128:i-121} \\ k_{i-120:i-113} \\ k_{i-112:i-105} \\ k_{i-104:i-97} \end{bmatrix}\\&\quad \oplus \begin{bmatrix} S(k_{i-24:i-17}) \oplus \mathsf {RC}_{i/128}\\ S(k_{i-16:i-9})\\ S(k_{i-8:i-1})\\ S(k_{i-32:i-25}) \end{bmatrix} \end{aligned}$$

and for the remaining subsequences $k_{i+32:i+127}$, the formula is simply $k_j \leftarrow k_{j-128} \oplus k_{j-32}$ for $j\in \{i+32, \dots , i+127\}$.

In the case of AES-192, the sequence $k_{0:1663}$ is derived in a similar fashion from the encryption key $k_{0:191}$. Let $i \in \{192, 384, \dots , 1536\}$. For the subsequences $k_{i:i+31}$, the formula is

$$\begin{aligned}&\begin{bmatrix} k_{i:i+7} \\ k_{i+8:i+15} \\ k_{i+16:i+23} \\ k_{i+24:i+31} \end{bmatrix} \leftarrow \begin{bmatrix} k_{i-192:i-185} \\ k_{i-184:i-177} \\ k_{i-176:i-169} \\ k_{i-168:i-161} \end{bmatrix}\\&\quad \oplus \begin{bmatrix} S(k_{i-24:i-17}) \oplus \mathsf {RC}_{i/192}\\ S(k_{i-16:i-9})\\ S(k_{i-8:i-1})\\ S(k_{i-32:i-25}) \end{bmatrix} \end{aligned}$$

and for $k_{i+32:i+191}$, the formula is similarly $k_j \leftarrow k_{j-192} \oplus k_{j-32}$ for $j\in \{i+32, \dots , i+191\}$.

In the case of AES-256, the key sequence is $k_{0:1991}$ and the encryption key is $k_{0:255}$. Let $i \in \{256, 512, \dots , 1792\}$. The subsequences $k_{i:i+32}$ for $1 \le i \le 14$ are derived with:

$$\begin{aligned}&\begin{bmatrix} k_{i:i+7} \\ k_{i+8:i+15} \\ k_{i+16:i+23} \\ k_{i+24:i+31} \end{bmatrix} \leftarrow \begin{bmatrix} k_{i-256:i-249} \\ k_{i-248:i-241} \\ k_{i-240:i-233} \\ k_{i-232:i-225} \end{bmatrix}\\&\quad \oplus \begin{bmatrix} S(k_{i-24:i-17}) \oplus \mathsf {RC}_{i/256}\\ S(k_{i-16:i-9})\\ S(k_{i-8:i-1})\\ S(k_{i-32:i-25}) \end{bmatrix} \end{aligned}$$

Additionally, the subsequences $k_{i+128:i+160}$ are derived with yet another formula:

$$\begin{aligned}&\begin{bmatrix} k_{i+128:i+135} \\ k_{i+136:i+143} \\ k_{i+144:i+151} \\ k_{i+152:i+159} \end{bmatrix} \leftarrow \begin{bmatrix} k_{i-128:i-121} \\ k_{i-120:i-113} \\ k_{i-112:i-105} \\ k_{i-104:i-97} \end{bmatrix}\\&\quad \oplus \begin{bmatrix} S(k_{i+96:i+103}) \\ S(k_{i+104:i+111})\\ S(k_{i+112:i+119})\\ S(k_{i+120:i+127}) \end{bmatrix} \end{aligned}$$

and for $k_{i+32:i+255}$, the formula is similarly $k_j \leftarrow k_{j-256} \oplus k_{j-32}$ for $j\in \{i+32, \dots , i+255\}$.

From a serial circuit perspective, these operations can be easily executed. We simply see all these updates in terms of two basic operations: sxor (S-box and XOR) and kxor (key bit and XOR). In the former, a byte value from particular position is updated by XORing itself with the output of S-box, where the input of S-box is chosen from the last column. In the latter, a bit value at particular position only needs to be XORed with another bit.

## Bit-serial circuit preliminaries

### Pipelines

At the core of our circuit lies two clearly separated pipelines that share some components, i.e., mainly S-box. Those pipelines are initially formed by a series of connected D flip-flops without asynchronous reset or enable signals (which we denote by FF^2^). Large sequences of flip-flops are employed in our pipelines, and hence, we use the sequence $\mathsf {FF}_{0:\ell -1}$ to denote an $\ell$-bit pipeline. A pipeline is constructed such that the output of $\mathsf {FF}_j$ is connected to the input of $\mathsf {FF}_{j-1}$ for $j \in \{1, 2, \dots , \ell -1\}$. Therefore, bits enter to the pipeline through $\mathsf {FF}_{\ell -1}$, visit flip-flops in descending order and exit from $\mathsf {FF}_0$. If no operation were to be executed except this natural shifting, a bit would spend $\ell$ clock cycles in the pipeline. The two pipelines of our design are:

Data pipeline: $\mathsf {FF}_{0:127}$ are arranged so that bits move from right to left in a byte, and column-wise bottom-to-top fashion. Each bit enters the pipeline from $\mathsf {FF}_{127}$ and exits from $\mathsf {FF}_0$, as shown in Fig. 2.
Key pipeline: $\mathsf {FF}_{0:255}$ are arranged in the same byte-columnar fashion as the data pipeline. As AES-128 and AES-192 require less than the 256 flip-flops, we bypass some parts of the pipeline for AES-128, AES-192.

However, we use the same variables $\mathsf {FF}$ for two pipelines, to which pipeline (either data or key) we refer to will be clear from the context. Below, we explain how we evolve the design of the pipeline so that it supports all the operations AES requires. They also explain which FF units must be replaced by a scan flip-flop.

### Primal pipeline operations

- Swap is the basic operation that allows exchanging bits stored in two flip-flops in a pipeline, if activated. Let us explain the working mechanism of swaps in more detail. For the sake of the example, let (*a*, *b*) denote a swap operation in the pipeline. Suppose that bit $x_a$ is stored in $\mathsf {FF}_a$, and bit $x_b$ is stored in $\mathsf {FF}_b$ in the current clock cycle. If the swap operation is inactive, $\mathsf {FF}_{a-1}$ and $\mathsf {FF}_{b-1}$ will store $x_a$ and $x_b$, respectively, in the next clock cycle. However, if we activate the swap operation, then these two flip-flops will store $x_b$ and $x_a$, respectively (with swapped order). On the netlist, this can be realized by adding MUXes at the input of $\mathsf {FF}_{b-1}$ and $\mathsf {FF}_{a-1}$, and wiring the outputs of $\mathsf {FF}_a$ and $\mathsf {FF}_b$ to both MUXes^3^. One can add many such swap operations to the pipeline. This idea was introduced by Banik et al. [16], and we extend the use of Swap s particularly for all AES versions to perform ShiftRows operation and column rotation required during the key expansion.
- Overwriting is an operation primitive that allows to load a different result to a set of registers during a particular cycle. In the netlist, this is constructed as a set of MUXes placed before the inputs of the registers whose value is to be overwritten by a different signal. Whether or not the value is overwritten is determined by a selector. For instance, this operation is used to load the results from S-box and MixColumns circuits to data pipeline.
- Bypassing is an operation primitive that allows to shorten a pipeline path, skipping a predetermined number of registers. On the netlist, this is realized by a simple MUX. This operation will be used to disable large portion of the key pipeline that is not used during AES-128 and AES-192.

### Components

Apart from the pipelines, the circuit includes a controller circuit, a combined circuit for S-box/inv-S-box and three MixColumns components. We readily borrow the smallest implementations of these primitives from the state of the art.

**Fig. 1:**
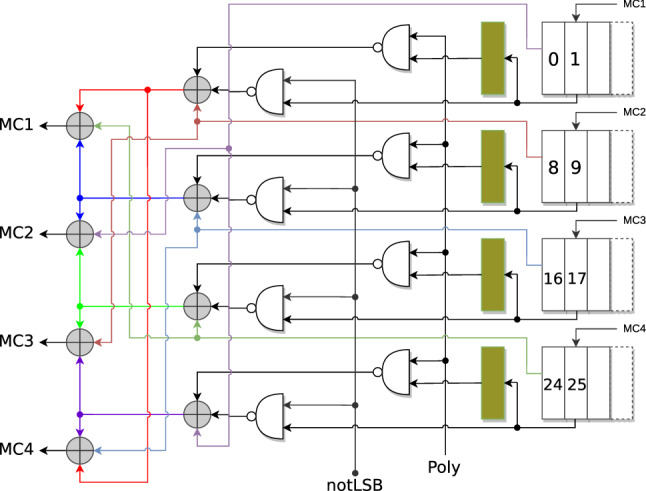
The MixColumns circuit of [4]

The MixColumns circuit we employ is from the Jean et al. which costs 8 XOR s, 8 NANDs and 4 enabled flip-flops (EFF s) [4] (see Fig. 1). This circuit reads 4-bit input in 1-bit per row fashion and outputs 4-bit output each clock cycle. Hence, processing one full column takes 8 clock cycles.

The circuit is designed to operate in a bit-serial fashion over each column. Note that since the multiplication by 2 (or 3) of any byte in the AES finite field depends on the value of the most significant bit of the concerned byte, one needs to store this MSB in a separate flip-flop when performing such a bit-serial multiplication. To ensure that the circuit operates seamlessly in the 8 cycles (say numbered from *t* to $t+7$), it is necessary to store the MSB of each byte of the current column at cycle $t-1$, when it occupies flip-flops 1, 9, 17 and 25, into the auxiliary dark green colored flip-flops shown in Fig. 1. This way from cycle *t* onward to $t+7$, the auxiliary flip-flops always store the MSB of each of the bytes of the column over which the MixColumns operation is to be performed. Now, if the single bit signal Poly takes the sequence of values 00011011 (0x1B) and the signal notLSB takes the values 11111110 (0xFE) in each of the cycles *t* to $t+7$, then it is trivial to see that the circuit faithfully outputs each of the 8 mixcolumn output bits in cycles *t* to $t+7$ serially.

The S-box implementation has been taken from Maximov and Ekdahl’s recent work, where the authors give the smallest known S-box occupying 253.35 GE (*bonus* of Table 5 of [17]). The circuit includes a combination of S-box and inverse S-box, the latter of which is required during decryption in the data path.

Finally, the control logic consists of a 11-bit counter, whose 4 upper bits are used for determining the round that is being executed. Since executing a single round takes exactly 128 clock cycles, the lower 7 bits are used to determine the phases within each round. Then, the controller administers every component in the circuit, mainly activating swaps, overwriting and bypassing operations based on the correct phase and round values. In total, the circuit on the high-level view can be seen as combination of 1) the data pipeline (with built-in MixColumns circuits), 2) the key pipeline, 3) the shared bidirectional S-box and 4) the controller.

### Hardware API and input formats

The AES architecture we introduce in the following section is a clocked serial one, having thus a 1-bit data path. We therefore have a 1-bit input port for the key input, 1-bit input port for the data input, a 2-bit selector for the AES version (AES-128/192/256), a 1-bit selector for the mode (i.e., direction of encryption/decryption), a clock signal Clk and a synchronous active-low reset signal Rst. The output consists of a 1-bit data port DataOut which carries the final result of ciphertext during encryption (or plaintext for decryption) and 1-bit control signal Done which flags that the final result will become available in the following 128 clock cycles. The latter control signal allows our design to be immediately used by an external mode of operation without having to count the number of clock cycles.

The bit string *d* denotes either the data (plaintext or the ciphertext depending on the direction of the operation), and we parse it as the bit sequence $d_{0}, \dots , d_{127}$, where $d_0$ corresponds to the leftmost bit of *d*. The data are always loaded during the first 128 clock cycles after reset regardless of the key length. Furthermore, the sequence is loaded in ascending order, i.e., starting from $d_0$, regardless of direction of the operation.

We further use the large sequence $k_{0},\dots , k_{128\cdot r+127}$ to denote the whole sequence of key bits derived with the key expansion algorithm during the complete encryption operation, where *r* denotes the number of rounds, i.e., $r \in \{10,12,14\}$. The key always loads in the first 128 (resp. 192, 256) clock cycles for AES-128 (resp. AES-192, AES-256) regardless of the direction of the operation. However, the order of loading and the particular subsequence to be loaded depends on the key length and the direction of the operation. This is given in Table 2.

**Table 2:** The order and time for loading key and data bits

Key len.	Oper.	Bit ordering	Cycles
128	Enc	$k_{0:127}$	[0, 127]
192	Enc	$k_{0:191}$	[0, 191]
256	Enc	$k_{0:255}$	[0, 255]
128	Dec	$k_{1280:1407}$	[0, 127]
192	Dec	$k_{1536:1663} \mathbin \Vert k_{1472:1535}$	[0, 191]
256	Dec	$k_{1792:1919} \mathbin \Vert k_{1664:1791}$	[0, 255]
(all)	Enc	$d_0, d_1, \dots , d_{127}$	[0, 127]
(all)	Dec	$d_0, d_1, \dots , d_{127}$	[0, 127]

Each round takes exactly 128 clock cycles to execute, and therefore, both encryption and decryption operations take 1408, 1664 and 1920 clock cycles for AES-128, AES-192 and AES-256, respectively. In the last 128 clock cycles, the ciphertext (for encryption) or plaintext (for decryption) becomes available and the order in which the output bits are produced follows the same order as the input.

## Data pipeline

Before moving on to the full-fledged details of our data pipeline, let us briefly explain the intuition behind our pipeline-based design, which similarly applies to the key scheduling in Sect. 5. We first treat each bit position individually and consider the set of operations a particular bit is supposed to pass through until its next round value is produced, i.e., each bit needs to execute AddRoundKey, SubBytes, ShiftRows, MixColumns by carefully interacting with other bits. It is clear that the combination of operations depends on the position of the bit, and they are not same for all. Moreover, MixColumns and SubBytes operations create dependence among bits, and we have to ensure that the correct choice of bits is forwarded to these units for executing together, not separately. For example, SubBytes operates at byte level (i.e., 8-bit input and 8-bit output) and each individual bit needs to appear at the correct input port of the S-box, and also each byte from the S-box output port must be written back into the pipeline in the appropriate fashion. Similarly, carefully chosen set of bits need to appear at 4-bit ports of MixColumns at the right time. Hence, the pipeline acts as a highly flexible storage unit, in which bits are dynamically moved around and driven into the input ports of each SubBytes, ShiftRows and MixColumns in 128 clock cycles. In order to move the bits around cheaply (in terms of extra gates required), we rely heavily on swap operations.

For our circuit, the data pipeline could be seen as a combination of fundamental operations based on the primal ones explained above:

- swap-32: Operation that performs a swap between two bits in adjacent columns (according to the classical state notation *St*), with a distance of 32 bits between them (thus the bits in question are in the same row) in 8 clock cycles. This operation is used to perform the ShiftRows for the second and fourth rows, where we have a rotation by one and by three bytes, respectively. For example, if $St_{i,j}$ ($i,j \in [0,3]$) denotes the *i*, *j*-th byte in the $4\times 4$ state matrix, then the shiftrow operation on the 2nd row essentially requires the byte sequence $St_{1,0}$, $St_{1,1}$, $St_{1,2}$, $St_{1,3}$ shifts to $St_{1,1}$, $St_{1,2}$, $St_{1,3}$, $St_{1,0}$. Three swap-32 operations execute the required transformation in the following manner:
  $$\begin{aligned} \begin{aligned}&~[St_{1,0},~St_{1,1},~St_{1,2},~St_{1,3}]{\mathop {\rightarrow }\limits ^{\mathsf{swap32}}}\\&\quad [St_{1,1},~St_{1,0},~St_{1,2},~St_{1,3}] \\ \quad {\mathop {\rightarrow }\limits ^{\mathsf{swap32}}}&~ [St_{1,1},~St_{1,2},~St_{1,0},~St_{1,3}] {\mathop {\rightarrow }\limits ^{\mathsf{swap32}}}\\&\quad [St_{1,1},~St_{1,2},~St_{1,3},~St_{1,0}] \end{aligned} \end{aligned}$$
- swap-64: Operation that performs a swap between two bits in columns with a distance of 64 bits between them. This operation is used to perform the ShiftRows for the third and fourth rows, where we have a rotation by two and by three positions, respectively. For example, in the third row, two swap-64 operations execute the transformation in the following manner:
  $$\begin{aligned} \begin{aligned}{}[St_{2,0},~St_{2,1},~St_{2,2},~St_{2,3}]{\mathop {\rightarrow }\limits ^{\mathsf{swap64}}}&~[St_{2,2},~St_{2,1},~St_{2,0},~St_{2,3}] \\ {\mathop {\rightarrow }\limits ^{\mathsf{swap64}}}&~ [St_{2,2},~St_{2,3},~St_{2,0},~St_{2,1}] \end{aligned} \end{aligned}$$
- swap-96: Operation that performs a swap between two bits in columns with a distance of 96 bits between them. This operation is used to perform the ShiftRows for the fourth row, where we have a rotation by three positions. The fourth row operation is executed thus:
  $$\begin{aligned} \begin{aligned}&~[St_{3,0},~St_{3,1},~St_{3,2},~St_{3,3}]{\mathop {\rightarrow }\limits ^{\mathsf{swap96}}}\\&\quad [St_{3,3},~St_{3,1},~St_{3,2},~St_{3,0}] \\ \quad {\mathop {\rightarrow }\limits ^{\mathsf{swap64}}}&~ [St_{3,3},~St_{3,0},~St_{3,2},~St_{3,1}] {\mathop {\rightarrow }\limits ^{\mathsf{swap32}}}\\&\quad [St_{3,3},~St_{3,0},~St_{3,1},~St_{3,2}] \end{aligned} \end{aligned}$$
- S-box: Operation that performs the S-box of an input byte and overwrites the output to flip-flops with the resulting byte. When the input flip-flops are chosen as $\mathsf {FF}_{a:a+7}$ for some *a*, the output is written back to $\mathsf {FF}_{a-1:a+6}$ so that the pipeline rotation is taken into account. It is used to perform the SubBytes operation when applied to each byte in the data pipeline, applying once each 8 cycles.
- MixColumns: Operation that performs MixColumns of a given column taking two adjacent bits at a time to produce 4 output bits per cycle. It is used to perform the MixColumns of each of the four columns during an state update round and requires 8 cycles per columns to do so.

In what follows, we present the complete data path circuit for AES encryption and decryption.

**Fig. 2:**
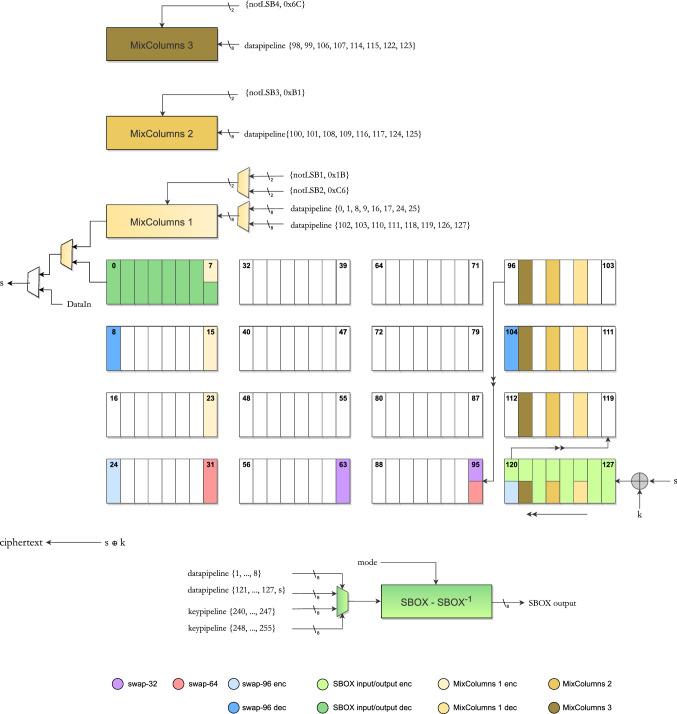
The data pipeline circuit. Note that each colored FF denotes a scan flip-flop (or equivalently flip-flops with a MUX at input). The flip-flops with multiple color indicate that there are multiple MUXes connected at their input

### Encryption

Let $\mathsf {round}$ denote the 4-bit counter for the number of rounds currently executed. We further use 7-bit counter $\mathsf {count}$ for the number of clock cycles executed from the beginning of each round. Namely, $\mathsf {round}$ is set to 0 at the beginning of the operation, and $\mathsf {count}$ is set to 0 at the beginning of each round. The $\mathsf {round}$ signal is incremented, and the $\mathsf {count}$ signal is reset after $\mathsf {count}$ reaches 127.

#### The S-box operation

Note that plaintext is pushed bit-wise into the circuit via the DataIn port, where it is XORed to the whitening key and loaded into the pipeline through $\mathsf {FF}_{127}$. Thereafter, the first operation to be performed each round is SubBytes. For this purpose, S-box is used in the data pipeline every eighth cycle during $\mathsf {count} \equiv 7 \bmod 8$. The S-box operation required in the key schedule function is used in a different cycle, that is $\not \equiv 7 \bmod 8$; therefore, this component can be shared between the data and key pipelines without any interruption, which will be further explained in Sect. 5. The elements involved in this operation can be seen in light green color in Fig. 2, including S-box and the 8 scan flip-flops involved in the process. The S-box reads the input from $\mathsf {FF}_{121:127} \mathbin \Vert (s\oplus k)$ (where $s \oplus k$ denotes the input wire of $\mathsf {FF}_{127}$ as shown in Fig. 2), and the output of the S-box is overwritten to $\mathsf {FF}_{120:127}$ in the following clock cycle. This operation is executed 16 times in each round to compute the entire SubBytes layer.

#### The ShiftRows operation

Note that each bit flowing out from $\mathsf {FF}_{120}$ has already undergone the S-box operation. Naturally, we now turn our attention to the ShiftRows operation.

ShiftRows is performed using the three swap operations as described above. Among them, swap-32 is located at (64, 96), i.e., swaps the contents of $\mathsf {FF}_{64}$, $\mathsf {FF}_{96}$ and loads them into $\mathsf {FF}_{95}$, $\mathsf {FF}_{63}$, respectively, in the following clock cycle. It is active during cycles $\mathsf {count} \in [8, 15] \cup [24,31] \cup [72, 79] \cup [104, 111]$ to perform the four adjacent swaps required during encryption. One might wonder why this works. Note that at cycle $\mathsf {count}=72$, the flip-flops $\mathsf {FF}_{64:71}$ store the intermediate value of the state byte $St_{1,0}$ and the flip-flops $\mathsf {FF}_{96:103}$ store the intermediate value of the state byte $St_{1,1}$. Thus, activating swap-32 over cycles $\mathsf {count} \in [72,79]$ simply helps execute the swap $St_{1,0}\leftrightarrow St_{1,1}$. The following swap-32 operations are also executed in the given cycles:

1. $\mathsf {count} \in [104,111]$ executes the swap $St_{1,0}\leftrightarrow St_{1,2}$.
2. $\mathsf {count} \in [8,15]$ of the **next** round executes the swap $St_{1,0}\leftrightarrow St_{1,3}$. This completes the ShiftRows operation in the 1st row.
3. $\mathsf {count} \in [24,31]$ of the **next** round executes the swap $St_{3,1}\leftrightarrow St_{3,2}$ required in the 3rd row.
4. This indicates that swap-32 is not executed in $\mathsf {count} \in [8,15]$ and $\in [24,31]$ of the very first round and only begins executing from $\mathsf {count}=72$ of the current round and ends at $\mathsf {count}=31$ of the next round. To capture this idea symbolically, we introduce the notation that swap-32 is actually executed over the cycles $\mathsf {count} \in [72,79] \cup [104,111] \cup [8,15]^{\pmb {+}} \cup [24,31]^{\pmb {+}}$. Here, $^{\pmb {+}}$ symbol indicates that execution of the operation overflows into the next round in the timetable.

This tells us an interesting fact: that a part of the ShiftRows operation of the current round is executed in the circuit in the numerically subsequent round. The challenge, therefore, from an engineering point of view, is to manage other operations like MixColumns and AddRoundKey, given that the ShiftRows operations are narrowly timed. Let us give the remaining two swap operations to complete ShiftRows. The swap-64 is located at (32, 96) and is active in cycles $\mathsf {count} \in [112, 119] \cup [16, 31]^{\pmb {+}}$. The swap-96 is located at (25, 121) and is active in cycles $\mathsf {count} \in \{127\} \cup [0, 6]^{\pmb {+}}$. It is not difficult to verify that these swaps faithfully execute the ShiftRows operation. These swaps are represented in purple, red and light blue colors, respectively, in Fig. 2.

As a final note, one might interpret our description above as if swap-96 (uses the bit stored at $\mathsf {FF}_{121}$) is being executed before S-box (overwrites its result to $\mathsf {FF}_{120}$). In order to correctly encrypt according to the AES specification, clearly SubBytes must precede ShiftRows. This dependence between the two operations is resolved in a rather subtle way, by ensuring that the swap operation actually takes its input from the output of the MUX placed at the input of $\mathsf {FF}_{120}$ (instead of the value stored at $\mathsf {FF}_{121}$). By doing so, it is guaranteed that the swapped bit comes from the output of the S-box, and the correct ordering between the operations SubBytes and ShiftRows is satisfied.

#### The MixColumns operation

We now turn our attention to MixColumns. Note that this component has been scheduled and placed near the circuit exit in order to leave as many cycles as possible for the previous operations to execute. In our circuit, the MixColumns operation of the current round also takes place in the subsequent round. The first such operation takes place in cycles $\mathsf {count} \in [0,7]$ of next round, where the MSB is stored to the internal flip-flops of the MixColumns circuitry in $\mathsf {count}=127$ of the current round as explained in Sect. 3.3. It is not difficult to see why these cycles are chosen. At cycle 0 of the next round, three bytes in the 1st column of the AES state have already had the ShiftRows operation performed on them. This is because in the following cycles of the current round:

1. $\mathsf {count} \in [72,79]$ executes the swap $St_{1,0}\leftrightarrow St_{1,1}$
2. $\mathsf {count} \in [112,119]$ executes the swap $St_{2,0}\leftrightarrow St_{2,2}$
3. At $\mathsf {count}=127$, the swap between the MSBs of $St_{3,0}\leftrightarrow St_{3,3}$ takes place between $\mathsf {FF}_{25}$ and $\mathsf {FF}_{121}$. As a result, at $\mathsf {count}=127$, the MSB of $St_{3,3}$ is available at the output of the MUX before $\mathsf {FF}_{24}$, and so stored in the internal flip-flop of the MixColumns circuitry as required. Thereafter, at every cycle $\mathsf {count}\in [0,6]$ of the subsequent round, each following bit of $St_{3,0}$ and $St_{3,3}$ is swapped in. This ensures that at cycles $\mathsf {count}\in [0,7]$, all the appropriate bits of $St_{3,3}$ occupy the $\mathsf {FF}_{24:25}$. Since the entire bytes $St_{0,0},~St_{1,1},$ and $St_{2,2}$ are already correctly placed in $\mathsf {FF}_{0:7}$, $\mathsf {FF}_{8:15}$ and $\mathsf {FF}_{16:23}$ at $\mathsf {count}=0$ of the next round, this ensures the MixColumns operation is faithfully executed, even though the entire byte $St_{3,3}$ is never actually stored in $\mathsf {FF}_{24 : 31}$ during $\mathsf {count}\in [0,7]$.

It is a matter of a simple arithmetic exercise to see that at $\mathsf {count}\in [32,39]\cup [64,71] \cup [96,103]$, the other 3 MixColumns operations for the remaining columns of the state matrix are also faithfully executed.

Now, let us make an observation on the locations where the MixColumns output is written back into the pipeline. Three of the output bits are naturally introduced into the pipeline at $\mathsf {FF}_7$, $\mathsf {FF}_{15}$ and $\mathsf {FF}_{23}$, which is in line with the continuously evolving nature of the pipeline. The most significant MixColumns output bit is introduced into the multiplexer after $\mathsf {FF}_0$, through which it becomes available at the *s* wire at the bottom right corner of Fig. 2. At this particular point, AddRoundKey is performed using the key bit produced by the key pipeline. Assuming that the key pipeline is able to produce the appropriate next round key bit at this cycle, the output of key XOR is written back in to the data pipeline at $\mathsf {FF}_{127}$ and so the AES round operations can be executed seamlessly. We will see in Sect. 5 how the key pipeline is engineered to produce key bits as required.

A cycle-by-cycle description of data pipeline encryption can be found in Fig. 4 following the above explanation. Note that the last AES round is arranged such that the MixColumns operation is skipped. The ciphertext bits are extracted from the port in the last 128 of the 1408 cycles used for encryption. Also, note that the data pipeline operations are the same for all 3 variants of AES, and the difference only arises from how the key pipelines are operated for each of them.

#### Alternate interpretation

**Fig. 3:**
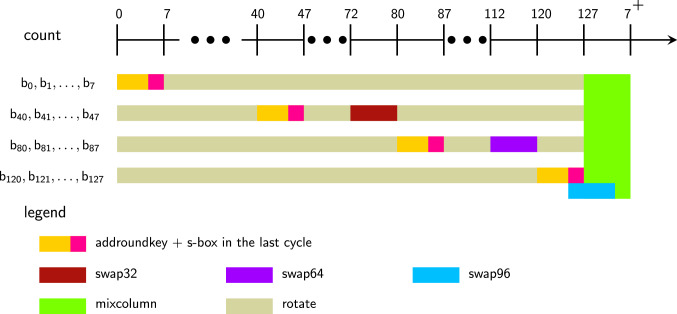
Another way to visualize the encryption data path from the point of view of the individual bits

**Fig. 4:**
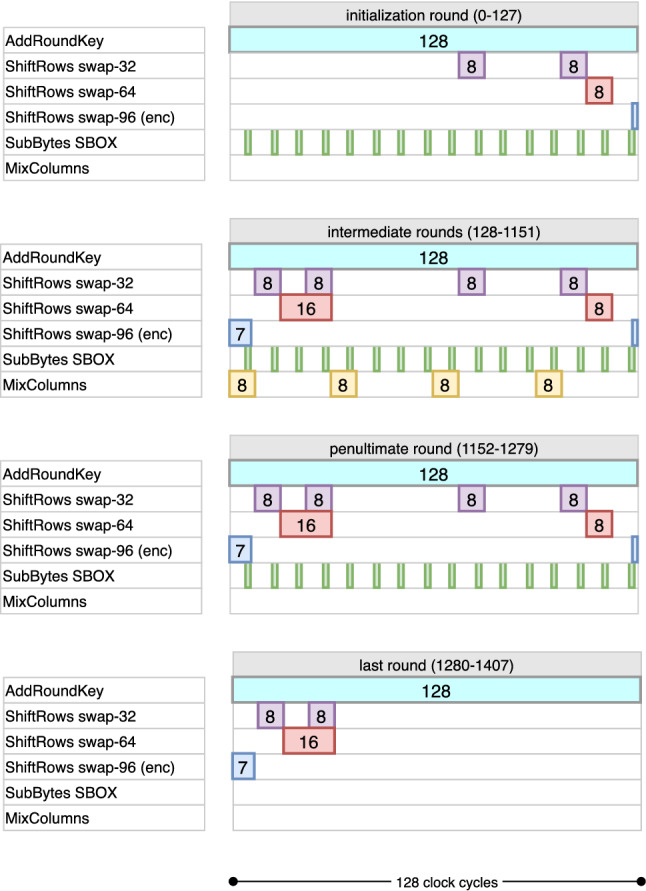
Cycle-by-cycle description of the data pipeline in encryption mode. The diagram only shows required activation cycles to obtain the correct output

To further explain the operations from the point of view of individual bits in the pipeline that are finally transformed through the MixColumns operation, please see Fig. 3. If we take *St* to be the state after S-box layer, then the output of MixColumns of the 1st column is essentially $2St_{0,0}+3St_{1,1}+St_{2,2}+St_{3,3}$, where $St_{0,0}=[b_0,b_1,\ldots ,b_7]$, $St_{1,1}=[b_{40},b_{41},\ldots ,b_{47}]$, $St_{2,2}=[b_{80},b_{81},\ldots ,b_{87}]$ and $St_{3,3}=[b_{120},b_{121},\ldots ,b_{127}]$. Note that MixColumns is performed on the flip-flops $\mathsf {FF}_{0:31}$ at cycles 0–7 of the next round. So the essential engineering in the encryption data path is to ensure that these aforementioned state bits arrive at these flip-flops in the above time frame after having been through the AddRoundKey and S-box layers. Note that each bit $b_i$ enters the pipeline after AddRoundKey operation and is sent through the S-box at the next possible count cycle which is $7\bmod 8$. After this, each bit has to go through one swap at a fixed time so that it is realigned in the pipeline and these bits are placed in the same column for the MixColumns operation. In the figure, we can see that the swap32, swap64, swap96 operations have been used on each set of bits judiciously so that this is possible. The reader can check that all the swap operations have been scheduled so that this alignment is achieved for all columns of the state in Fig. 4.

### Decryption

Our decryption circuit is slightly more complicated than the circuit of Jean et al. [4] on account of the fact that the authors used clock-gating to freeze the pipeline to gain extra cycles, allowing further reuse of circuit components. Therefore, the circuit in [4] requires almost the double number of clock cycles to perform AES decryption compared to ours, i.e., 2512 cycles instead of 1408, for all the three versions of AES.

Decryption requires us to perform the inverse operations in the reverse order. This change makes us move the S-box from the very beginning to the very end of the pipeline and also forces the inverse MixColumns to move from the left part of the circuit to the right part of it. Both modifications require us to place new swaps, because some of the previous positions cannot be reused. Inverse MixColumns is performed using the property that applying this operation four times results in the identity matrix [15]. Thus, we apply forward MixColumns three times, which will result in the inverse of the operation. Therefore, we add two new MixColumns logic components to the circuit. The paper [4] uses only one MixColumns circuit. As a result, to achieve the inverse MixColumns operation, each column has to be operated upon by this circuit a total of 3 times, which in turn increases circuit latency. Since we aim to keep the latency fixed at 1408 cycles, we employ this hardware redundancy, i.e., using 2 additional MixColumns logic circuit.

This results in an overhead of around 120 GE compared to bit-sliding circuit [4], but saves more than 1000 cycles for the decryption operation, which taking into consideration latency seems a reasonable trade-off.

#### Inverse ShiftRows operation

Initially, the ciphertext bits are again pushed into the pipeline through the DataIn port, where it is XORed with the decryption key and reintroduced into the pipeline through $\mathsf {FF}_{127}$. Thereafter, the first operations to be performed are the inverse ShiftRows (since the first decryption round does not execute inverse MixColumns), which involves rotating the *i*-th row toward the left by $4-i$ bytes for $i=1,2,3$. Thus, the only difference with the forward ShiftRows is that the 1st row is rotated left by the 3 bytes instead of 1 and the 3rd row is rotated left by 1 byte instead of 3. (The second row is rotated by 2 bytes in both operations.) Thus, the 1st and 3rd rows are transformed in the following manner:

$$\begin{aligned} \begin{aligned}&~[St_{1,0},~St_{1,1},~St_{1,2},~St_{1,3}]{\mathop {\rightarrow }\limits ^{\mathsf{swap96}}}\\&\quad [St_{1,3},~St_{1,1},~St_{1,2},~St_{1,0}] \\ \quad {\mathop {\rightarrow }\limits ^{\mathsf{swap64}}}&~ [St_{1,3},~St_{1,0},~St_{1,2},~St_{1,1}] {\mathop {\rightarrow }\limits ^{\mathsf{swap32}}}\\&\quad [St_{1,3},~St_{1,0},~St_{1,1},~St_{1,2}]\\~\\&\quad ~[St_{3,0},~St_{3,1},~St_{3,2},~St_{3,3}]{\mathop {\rightarrow }\limits ^{\mathsf{swap32}}}\\&\quad [St_{3,1},~St_{3,0},~St_{3,2},~St_{3,3}] \\ \quad {\mathop {\rightarrow }\limits ^{\mathsf{swap32}}}&~ [St_{3,1},~St_{3,2},~St_{3,0},~St_{3,3}] {\mathop {\rightarrow }\limits ^{\mathsf{swap32}}}\\&\quad [St_{3,1},~St_{3,2},~St_{3,3},~St_{3,0}] \end{aligned} \end{aligned}$$

The individual swaps are executed as follows:

1. swap-32 at cycle $\mathsf {count} \in [88, 95]\cup [120, 127] \cup [8, 15]^{\pmb {+}}$ $\cup [24, 31]^{\pmb {+}}$,
2. swap-64 at cycle $\mathsf {count} \in [112, 119] \cup [8, 23]^{\pmb {+}}$,
3. swap-96 at cycle $\mathsf {count} \in \{127\} \cup [0,6]^{\pmb {+}}$. In order to accommodate swap-96, we do not use the same locations from the encryption. Instead, we define a new location as (9, 105), i.e., swaps $\mathsf {FF}_9$ and $\mathsf {FF}_{105}$. The reason will become clear as we describe the remaining parts of the pipeline. Again it is not difficult to work out, by following the same logic described for the forward ShiftRows, that the above sequence of swaps correctly executes the inverse ShiftRows.

#### Inverse S-box operation

The S-box circuit that we use is also equipped to execute the inverse S-box operation, and so it fits seamlessly into our decryption data path. $\mathsf {FF}_{1:8}$ serve as the input ports, and the result is written back into $\mathsf {FF}_{0:7}$ in the following clock cycles. This operation is activated in cycles $\mathsf {count} \equiv 7 \bmod 8$ (represented in dark green in Fig. 2). Note that this does not create a conflict, because we ensure that the data bits entering $\mathsf {FF}_8$ have all been processed by the inverse ShiftRows operation.

**Fig. 5:**
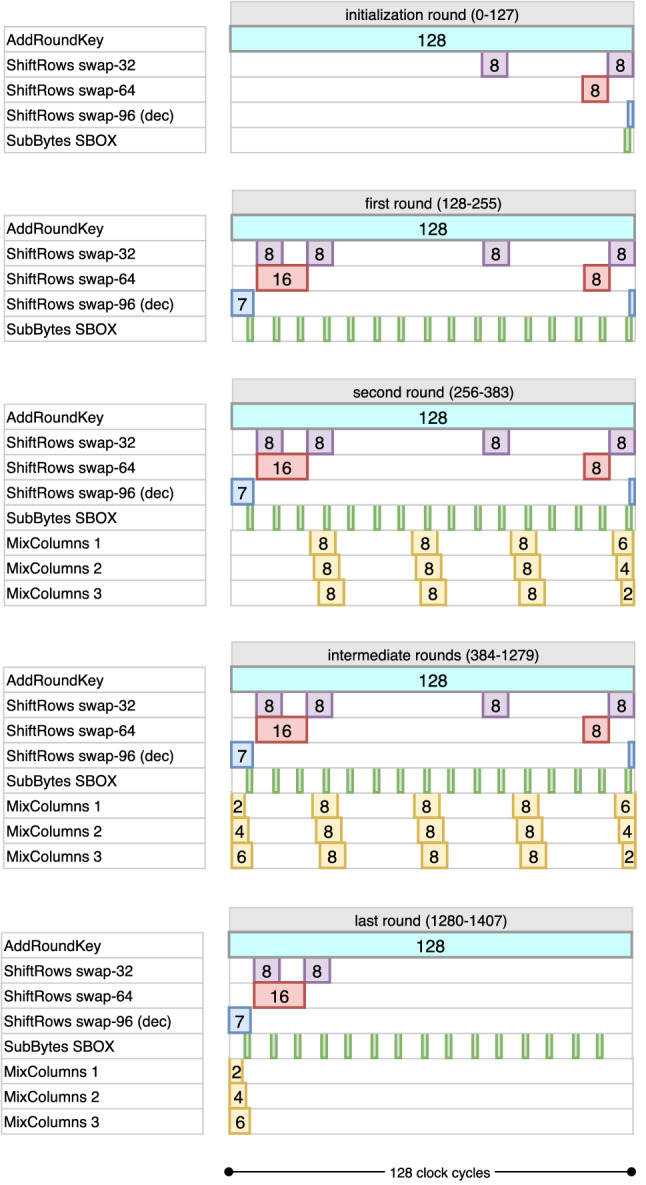
Cycle-by-cycle description of the data pipeline in decryption mode. The diagram only shows required activation cycles to obtain the correct output

**Fig. 6:**
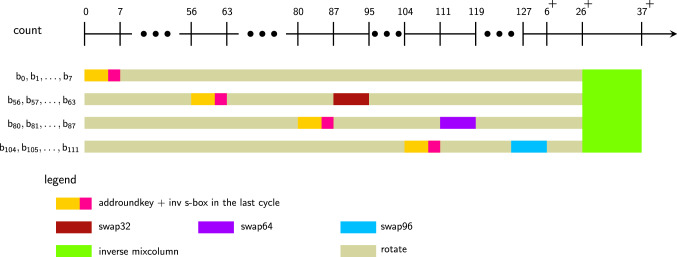
As described in Fig. 3, this figure represents an alternate way to visualize the decryption data path from the point of view of the individual bits that are passed through the first inverse-MixColumns layer

**Fig. 7:**
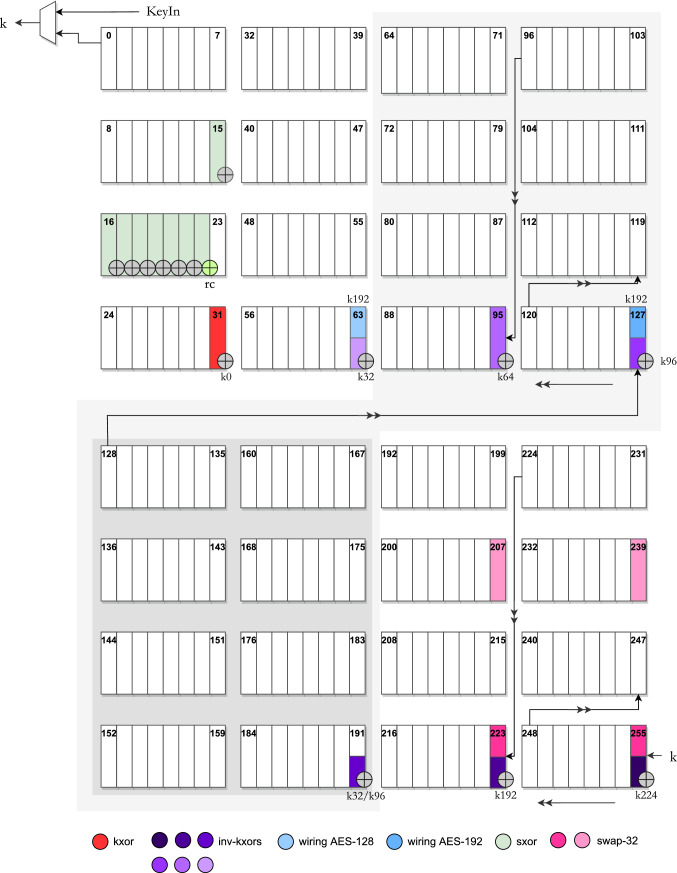
Key pipeline circuit

#### Inverse MixColumns operation

The data that leave the pipeline through $\mathsf {FF}_0$ are again added to the next decryption round key bit and reintroduced into the pipeline through $\mathsf {FF}_{127}$. This time, however, the first operation to be performed is the inverse MixColumns. From the explanation provided in Sects. 3.3 and 4, the following facts can be established:

- If the MixColumns operation is to be executed from cycles $\mathsf {count} \in [t, t+7]$, then the most significant bits of the bytes in each column need to be stored in auxiliary flip-flops at clock cycle $t-1$.
- For the MixColumns circuit to produce the correct output bits at any cycle $t_0\in [t,t+7]$, it is not necessary for all the bytes of the current column to be in place in the respective flip-flops. In fact the only condition that needs to be satisfied is that only 8 bits (2 bits from each byte) that are connected to MixColumns circuit must be ready.

The second fact allows us to implement 3 successive MixColumns operations in only 12 clock cycles in a cascaded manner. The idea is to use the locations $\mathsf {FF}_{102:103}$, $\mathsf {FF}_{110:111}$, $\mathsf {FF}_{118:119}$, $\mathsf {FF}_{126:127}$ as inputs to the 1st MixColumns circuit. This circuit is operated at $\mathsf {count} \in [26,33]$, and the outputs are written into $\mathsf {FF}_{101}$, $\mathsf {FF}_{109}$, $\mathsf {FF}_{117}$, $\mathsf {FF}_{125}$. We place the 2nd MixColumns circuit at a 2-bit distance with inputs $\mathsf {FF}_{100:101}$, $\mathsf {FF}_{108:109}$, $\mathsf {FF}_{116:117}$, $\mathsf {FF}_{124:125}$, with outputs $\mathsf {FF}_{99}$, $\mathsf {FF}_{107}$, $\mathsf {FF}_{115}$, $\mathsf {FF}_{123}$ such that it operates at $\mathsf {count} \in [28,35]$. Similarly, we place the 3rd MixColumns circuit at another 2-bit distance with inputs $\mathsf {FF}_{98:99}$, $\mathsf {FF}_{106:107}$, $\mathsf {FF}_{114:115}$, $\mathsf {FF}_{122:123}$ , with outputs $\mathsf {FF}_{97}$, $\mathsf {FF}_{105}$, $\mathsf {FF}_{113}$, $\mathsf {FF}_{121}$ such that it operates at $\mathsf {count} \in [30,37]$. It is easy to see that for all the 3 MixColumns circuits, the 2 most significant bits are in place during all the cycles they are executed. After that, it is really elementary to see that this executes the inverse MixColumns operation on the first column correctly. For the remaining columns, the operations are executed in count $\in [58, 69] \cup [90, 101] \cup [122, 127] \cup [0, 5]^{\pmb {+}}$. A cycle-by-cycle description of data pipeline decryption can be found in Fig. 5 following the above explanation. To further explain the operations from the point of view of individual bits in the pipeline that are finally transformed through the MixColumns operation, please see Fig. 6.

## Key pipeline

The key pipeline is in charge of producing a continuous stream of bits that will be consumed by the data pipeline, namely 1 bit of key each clock cycle, thus 128 bits each round. This must be fulfilled regardless of which functionality the circuit is executing.

Following the footsteps of Balli et al. [5], our key pipeline also consists of 256 flip-flops, denoted as a sequence $\mathsf {FF}_{0:255}$. The bits enter to pipeline through $\mathsf {FF}_{255}$ and exit from $\mathsf {FF}_0$. During AES-256, all flip-flops are active, but for AES-192 (resp. AES-128), we disable 64 (resp. 128) flip-flops so that the effective length of the pipeline matches the length of the key. This optionally disabled set of flip-flops is highlighted with gray background in Fig. 7. During initialization, the sequence of key bits is loaded starting from $\mathsf {FF}_{255}$.

Before giving the full cycle-by-cycle explanation of key pipeline, we first summarize our approach. During encryption operations, we are running the key schedule in the forward direction, i.e., the encryption key is loaded and the key expansion algorithm is run as defined. In contrast, during decryption, we start with the last round key (which we refer to as decryption key) and run the key scheduling in backwards. Therefore, it is clear that the pipeline should be able to perform key scheduling algorithm in both directions.

Let us first begin by explaining the forward key scheduling by using AES-128 as an example. As stated in Sect. 2.1.2, we can think key scheduling in terms of two operations: kxor and sxor. We follow the same notation and use $k_{0:1407}$ to represent the concatenation of all round keys in AES-128. For computing the second round key $k_{128:255}$ from the encryption key $k_{0:127}$, the first 32 bits $k_{128: 159}$ require sxor and the remaining 96 bits $k_{160:255}$ require kxor operation.

**Fig. 8:**
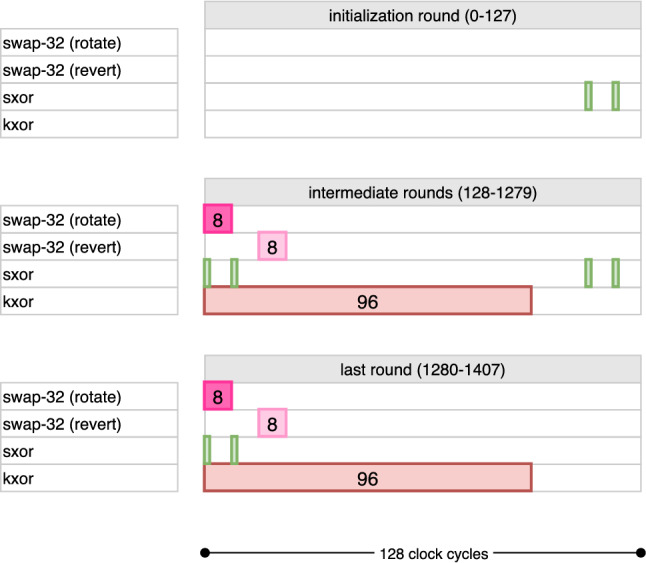
Cycle-level description of AES-128 key scheduling during encryption

We execute sxor operation 8-bit at a time as it uses S-box, which in turn is realized as 8-bit input, 8-bit output combinatorial circuit. Therefore, we perform sxor 4 times per key scheduling call, i.e., during 4 clock cycles per 128 clock cycles. More concretely, let us look at computation of $k_{128:159}$ from $k_{0:127}$ to understand what additional circuit is required. As far as the very first invocation of the key expansion is concerned, the equations listed in Sect. 2.1.2 lend themselves to:One can notice that the terms with the same colors correspond to bits of the same position from the previous and next round keys, if we consider keys in terms of 128-bit blocks. This essentially means that, in order to derive those bits of the next round key, all we need to do is to XOR a byte itself with the output of S-box, while ensuring that the input of S-box is correctly wired to receive values $k_{104:111}$, $k_{112:119}$, $k_{120:127}$, $k_{96:103}$, respectively. In Fig. 8, the update XOR circuitry is positioned at the input ports of $\mathsf {FF}_{15:22}$, and the S-box inputs are read from $\mathsf {FF}_{248:255}$. Except the last of the four equations given above, the additional byte inputs naturally appear at $\mathsf {FF}_{248:255}$, while the updated byte resides in $\mathsf {FF}_{216:223}$. Therefore, S-box receives its input from $\mathsf {FF}_{248:255}$. In order to temporarily relocate the last byte $k_{96:103}$ into $\mathsf {FF}_{248:255}$ (which would otherwise be located at $\mathsf {FF}_{216:223}$, while $k_{24:31}$ is stored in $\mathsf {FF}_{16:23}$), we use swap operations. Yet another swap operation is introduced to revert back to the original ordering after S-box operation is complete.

The details regarding the bits with kxor operation are much simpler, as they only require XORing bits. For instance, if we take a look at one of the updates (out of 96 bits), $k_{160} \leftarrow k_{32} \oplus k_{128}$, it is clear that, similar to sxor operation, two bits sharing the same position between different round keys are connected with XOR of another value. This extra value always resides by a distance of 32. Therefore, when a bit that needs an update arrives to the exit of the pipeline, i.e., $\mathsf {FF}_0$, then the required extra bit resides in $\mathsf {FF}_{32}$. Therefore, a single XOR gate at the input of $\mathsf {FF}_{31}$ is sufficient to perform this operation as marked with red in Fig. 7. And lastly, the round constant addition is performed through a lookup table.

For decryption, we will execute the key scheduling in the reverse order, which also means that we need the swap the order of execution between sxor and kxor. For sxor, we will use the same circuitry, but for kxor, we introduce few additional gates and refer to this operation with inv-kxor. Unlike kxor, inv-kxor must be executed in parallel (in 32 clock cycles), in order to ensure that the values required by S-box become available.

In what follows, we present the cycle-by-cycle details of key expansion circuit and the corresponding explanations for each version and mode in an incremental fashion from AES-128 encryption to AES-256 decryption.

In order to simplify the explanation, we first introduce two additional artificial counters. Let $\mathsf {round_{key}}$ denote the 4-bit counter for the number of key expansion calls made during AES execution. Let $\mathsf {count_{key}}$ be an 8-bit counter for the number of clock cycles passed during the expansion. These two counters are slightly different than those $\mathsf {round}$ and $\mathsf {count}$ introduced in Sect. 4.1, because the former pair is synchronized with key scheduling, whereas the latter pair is synchronized with the encryption/decryption rounds. In circuit, these counters are computed with a combinatorial circuit from $(\mathsf {round}, \mathsf {count})$ instead of using extra registers. Note that $\mathsf {count_{key}}$ counts up to 128, 192, 256 for AES-128, AES-192 and AES-256, respectively.

### AES-128 Encryption

For AES-128 encryption and decryption, $\mathsf {count_{key}}$ (counts up to 127) and $\mathsf {round_{key}}$ (counts up to 10) match $\mathsf {count}$ and $\mathsf {round}$ precisely. The four middle columns, from $\mathsf {FF}_{64}$ to $\mathsf {FF}_{191}$, are wired out of the pipeline, utilizing the light blue wiring in Fig. 7, so that only half of the available flip-flops are active. In other words, the output bit of $\mathsf {FF}_{192}$ is wired to the input of $\mathsf {FF}_{63}$ through a MUX.

At the beginning, both counters are set to 0. AddRoundKey operation already uses the key that is already being loaded for the first 128 clock cycles.

The S-box is activated four times per round on clock cycles 0, 8, 112 and 120. It uses the flip-flops $\mathsf {FF}_{248:255}$ as input, and the result of $\textsf {S-box} (\mathsf {FF}_{248:255}) \oplus \mathsf {FF}_{16:23}$ is stored in $\mathsf {FF}_{15:22}$ in the next clock cycle. The round constant is added in a bit-wise fashion through an extra XOR gate at the input of $\mathsf {FF}_{23}$ before S-box, and it computed through a lookup table. XOR gates belonging to S-box operation are represented in dark green in Fig. 7. The XOR gate that handles the round constant addition is represented in light green. The positions for S-box operation are chosen as $\mathsf {FF}_{15:22}$ so that we can execute this operation as early as possible, i.e., as soon as the additional byte appears at $\mathsf {FF}_{248:255}$. The only exception is the last execution of S-box operation (for each round). During clock cycles $\mathsf {count_{key}} \in [0, 7]$, we use the swap-32 to temporarily relocate the byte $k_{96:103}$ into $\mathsf {FF}_{248:255}$ so that S-box can get its input from $\mathsf {FF}_{248:255}$. This repositioning is reverted at clock cycle $\mathsf {count_{key}} \in [16, 23]$ using yet another swap-32. Both swaps are represented in Fig. 7 in dark and light pink, respectively.

During clock cycles $\mathsf {count_{key}} \in [0, 95]$ of each round, the kxor operation is active and computes the last 96 bits of the new round key on the fly. This is done XORing the output bit of the pipeline to the input of $\mathsf {FF}_{31}$. The full timetable of operations is given for AES-128 in Fig. 8.

### AES-128 Decryption

**Fig. 9:**
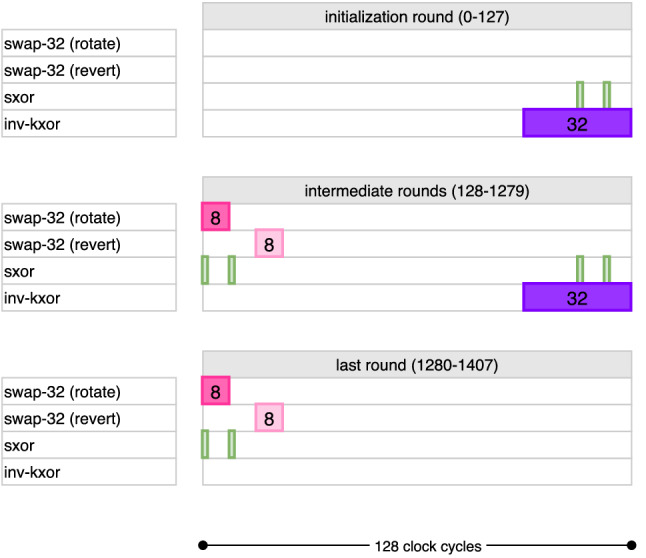
Cycle-level description of AES-128 key scheduling during decryption

**Fig. 10:**
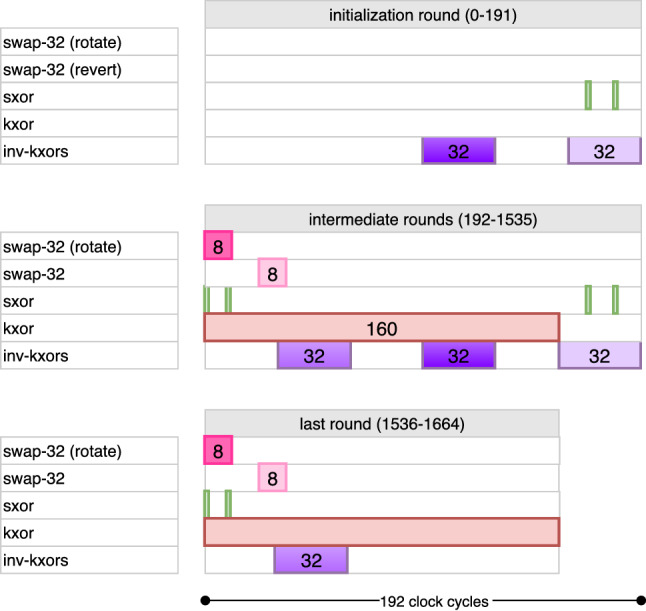
Combined cycle-by-cycle description of the key pipeline for AES-192. kxor (resp. inv-kxor) is active only during encryption (resp. decryption), whereas swap-32 and sxor are active in both

For decryption, we perform the key scheduling in the reverse order. Similarly, we use two counters $\mathsf {count_{key}}$ (counts up to 127) and $\mathsf {round_{key}}$ (counts up to 10). The counters are reset to 0 just before the key loading starts.

During decryption, the gates belonging to kxor operation are idle, as we introduce new set of XOR gates to perform inv-kxor. The last 96 bits of the previous round key are computed during cycle $\mathsf {count} \in [96, 127]$ using the two darkest and the lightest purple inv-kxor represented in Fig. 7. These are, namely the three XOR gates, at the inputs of $\mathsf {FF}_{63}$, $\mathsf {FF}_{223}$, $\mathsf {FF}_{255}$, where the additional values to these gates come from the outputs of $\mathsf {FF}_{32}$, $\mathsf {FF}_{192}$, $\mathsf {FF}_{224}$, respectively. This allows us to perform the whole operation in 32 cycles instead of 96 and more importantly lets us compute the necessary byte values for the S-box operation beforehand without changing its original position, namely $\mathsf {FF}_{248:255}$ as input to S-box and $\mathsf {FF}_{15:22}$ for storing the result.

Similarly, the round constant is added before $\mathsf {FF}_{22}$ through a lookup table.

Finally, rxor cycles are reversed to apply the XOR in the opposite order. The rest of the operations remain unchanged and similar to AES-128 encryption. A cycle-by-cycle description of key pipeline for AES-128 can be found in Fig. 9 following the above explanation in Fig. 10.

### AES-192 Encryption

For AES-192 encryption, $\mathsf {count_{key}}$ counts up to 191 in order to match the key length, and $\mathsf {round_{key}}$ counts up to the number of key expansion calls/rounds (i.e., up to 9).

For 192-bit key scheduling, 64 flip-flops are wired out, from $\mathsf {FF}_{128}$ to $\mathsf {FF}_{191}$, denoted as dark wiring component in the circuit in Fig. 7. In other words, the output of $\mathsf {FF}_{192}$ is wired to $\mathsf {FF}_{127}$. Because the key expansion algorithms treat key blocks regardless of their original size, e.g., 128 to 256, the same operations that we defined are readily usable in this version too.

At initialization, both counters are reset to 0. The next 192 clock cycles are used for loading the key into the pipeline. For this version, the S-box is active on clock cycles $\mathsf {count_{key}} \in \{ 0, 8, 176, 184\}$. With this operation, 32 bits of the next block of key are derived. The round constant is added through a lookup table as before.

The kxor operation is active for the rest of the bits in the block. This essentially takes 160 clock cycles to complete. Therefore, $\mathsf {count_{key}} \in [0, 159]$ produces these remaining bits. The repositioning operations, i.e., swap-32 and the restoring swap-32, remain unmodified, following the same idea in AES-128 encryption. In summary, the difference between AES-128 and AES-192 is handled through changing how the key counters are computed. As stated before, the round keys, which are 128-bit blocks, are continuously consumed by the data pipeline. The combined (both encryption and decryption) timetable of operations is given in Fig. 8. The full timetable of operations is given in Fig. 10.

### AES-192 Decryption

AES-192 decryption presents the most challenging part of the key pipeline, because this mode and version suffer the most from the lack of synchronization between the data path and the key path. The counter $\mathsf {count_{key}}$ counts up to 191, and $\mathsf {round_{key}}$ counts up to 9 as before.

Our main approach is similar to AES-128 decryption in that 1) we reuse S-box operations and a lookup table for round constant addition and 2) add necessary inv-kxor gates to handle the rest. The latter operation must be completed before we can move on to sxor, because of the dependency between the key bits.

The inv-kxor operation must be applied for the remaining 160 bits of key. For this, we use three different time slots and gate combinations. First, during $\mathsf {count_{key}} \in [32, 63]$, the two inv-kxor circuitries compute $\mathsf {FF}_{224} \oplus \mathsf {FF}_{192}$ and $k \oplus \mathsf {FF}_{224}$ (with *k* being the pipeline input) and load them into $\mathsf {FF}_{223}$ and $\mathsf {FF}_{255}$ in the following clock cycle, respectively. Secondly, during $\mathsf {count_{key}} \in [96, 127]$, the two inv-kxor circuitries compute $\mathsf {FF}_{96} \oplus \mathsf {FF}_{64}$ and $\mathsf {FF}_{192} \oplus \mathsf {FF}_{96}$ and load them into $\mathsf {FF}_{95}$ and $\mathsf {FF}_{127}$ in the following clock cycle, respectively. And lastly, during $\mathsf {count_{key}} \in [160, 191]$, the single inv-kxor circuitry computes $\mathsf {FF}_{64} \oplus \mathsf {FF}_{32}$ and loads it into $\mathsf {FF}_{63}$ in the following clock cycle. The gates and connections related to inv-kxor are marked with dark purple color in Fig. 7.

Key desynchronization requires that we also change the output port for receiving the round key bits. Namely, every 128 clock cycles, we shift among $\mathsf {FF}_0$, $\mathsf {FF}_{192}$ and $\mathsf {FF}_{64}$, in given order, for reading the key bit into AddRoundKey. This is realized through a MUX, which is not shown in Fig. 7. The full timetable of operations is given in Fig. 10.

### AES-256 Encryption

For AES-256 variant, $\mathsf {count_{key}}$ counts up to 255, and $\mathsf {round_{key}}$ counts up to 8. All flip-flops in the pipeline are active. Performing AES-256 key scheduling is quite similar to AES-128, with the exception that sxor operation needs to be applied 8 times instead of 4.

The sxor operation is active on clock cycles $\mathsf {count_{key}} \in \{0, 8, , 240, ,248\} \cup \{ 112, 120, 128, 136\}$. Here, the second set corresponds to the key update in the fifth column of 256-bit key block, as explained in Sect. 2.1.2. In the first set, we compute $\textsf {S-box} (\mathsf {FF}_{248:255}) \oplus \mathsf {FF}_{16:23}$ and load the result into $\mathsf {FF}_{15:22}$ in the following clock cycle, as done in AES-128 and AES-192. However, for the second set, we need to take into account that there is not column rotation, and hence, the value to be loaded into $\mathsf {FF}_{15:22}$ becomes $\textsf {S-box} (\mathsf {FF}_{240:247}) \oplus \mathsf {FF}_{16:23}$. We handle this by an additional 8-bit MUX in front of S-box, so that we can choose which input is used by S-box. The round constant is again computed through a lookup table.

**Fig. 11:**
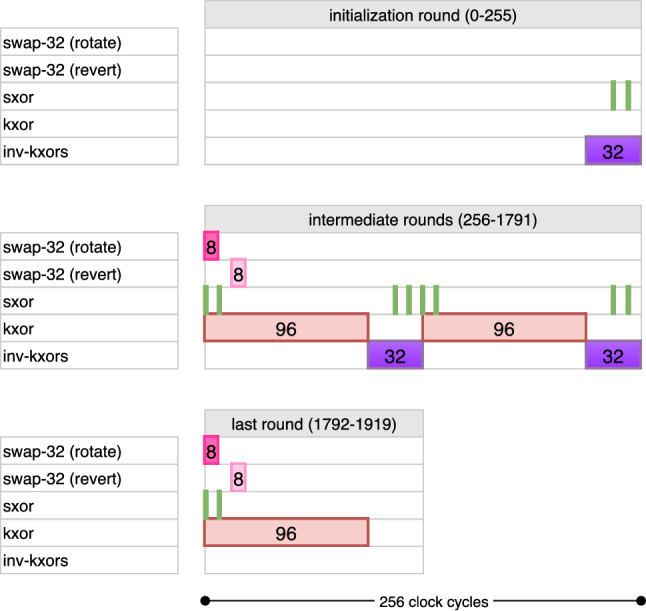
Combined cycle-by-cycle description of the key pipeline for AES-256. kxor (resp. inv-kxor) is only run during encryption (resp. decryption), while other operations are active in both directions

The kxor operation works exactly same and is repeated for the remaining 192 bits, in two disjoint sets $[32, 127] \cup [160, 255]$. The combined timetable of operations is given in Fig. 11.

**Table 3:** The area, power, throughput and latency measurements of our combined AES-128/192/256 architecture

Library	Area	Power ($\mu$W)		Area	$\mathsf {TP_{max}}$	Latency
	(GE)	@10MHz	@100KHz	$\mu$ $m^2$	(Mbps)	(cycles)
stm90	2268	153.60	2.80	9957.43	10.86	1408/1664/1920
umc90	2851	104.30	22.68	8940.74	11.56	1408/1664/1920
tsmc90	2905	75.23	1.73	8199.07	13.61	1408/1664/1920
nan45	3401	244.47	181.46	2714.26	36.36	1408/1664/1920
nan15	3835	29.08	10.52	753.99	259.31	1408/1664/1920

$\mathsf {TP_{max}}$ denotes the maximum throughput when the circuit is executing AES-128 encryption. Latency is given for all AES shades ordered by key length ascending order. Nangate 15nm/45nm and TSMC 90nm standard cell libraries are denoted with mnemonics nan15/nan45 and tsmc90. The STM 90nm and UMC 90nm libraries are denoted with mnemonics stm90/umc90

**Table 4:** The area, power, throughput and latency measurements of our stand-alone AES-192 and AES-256 implementations

Stand-alone	Library	Area	Power ($\mu$W)		Area	$\mathsf {TP_{max}}$	Latency
		(GE)	@10MHz	@100KHz	$\mu$ $m^2$	(Mbps)	(cycles)
AES-256 e	stm90	1702	113.10	2.11	7472.46	12.53	1920
	umc90	2179	86.71	17.54	6832.56	12.60	1920
	tsmc90	2263	71.43	1.52	6387.80	16.02	1920
	nan45	2725	195.61	139.74	2174.82	47.50	1920
	nan15	3125	25.37	8.50	614.45	312.39	1920
AES-256 e/d	stm90	2004	151.30	2.65	8798.36	11.35	1920
	umc90	2551	101.40	20.64	8000.72	12.51	1920
	tsmc90	2622	74.78	1.66	7401.04	13.87	1920
	nan45	3086	223.41	161.74	2462.89	40.30	1920
	nan15	3528	28.12	9.74	693.58	276.23	1920
AES-192 e	stm90	1511	100.10	1.87	6634.99	12.77	1664
	umc90	1924	74.54	15.53	6034.45	13.11	1664
	tsmc90	1981	60.43	1.30	5589.76	16.16	1664
	nan45	2372	170.14	122.43	1892.86	42.90	1664
	nan15	2715	21.81	7.45	533.74	323.23	1664
AES-192 e/d	stm90	1906	145.50	2.52	8368.10	9.14	1664
	umc90	2331	88.78	18.69	7310.02	11.23	1664
	tsmc90	2439	63.89	1.46	6883.13	10.41	1664
	nan45	2806	202.73	148.54	2239.19	30.93	1664
	nan15	3157	24.81	8.76	620.69	266.74	1664

Encryption only mode is denoted as Enc, and Encryption–Decryption mode is denoted with Enc/Dec

### AES-256 Decryption

The backwards key scheduling of AES-256 is quite similar to AES-128 decryption case, where the counter $\mathsf {count_{key}}$ counts up to 255, and $\mathsf {round_{key}}$ counts up to 8.

We again readily use the sxor operation from AES-256 encryption variant. Namely, the 8 invocations of sxor remain exactly same.

The inv-kxor operation is active during $\mathsf {count_{key}} \in [96, 127] \cup [224, 255]$. This operation computes the values $\mathsf {FF}_{128} \oplus \mathsf {FF}_{96}$, $\mathsf {FF}_{96} \oplus \mathsf {FF}_{64}$ and $\mathsf {FF}_{64} \oplus \mathsf {FF}_{32}$ and loads them into $\mathsf {FF}_{127}$, $\mathsf {FF}_{95}$ and $\mathsf {FF}_{63}$, respectively, in the following clock cycles. These gates are also marked in dark purple in Fig. 7. The combined timetable of operations can be found in Fig. 11.

## Results and discussion

The circuit was first modeled in Python for preliminary testing and later implemented directly at register-transfer level (RTL) with a hardware description language (i.e., VHDL). This HDL implementation was initially tested using *Mentor Graphics Modelsim* simulation software against pre-computed test vectors, which only verifies the functional correctness. We then synthesized the circuit as ASIC by instructing *Synopsys Design Compiler* to do all-in-one optimization through compile_ultra setting against five different CMOS technology libraries, namely STM 90 nm, UMC 90 nm, TSMC 90 nm, Nangate 15 nm and Nangate 45 nm. We further verified the post-synthesis correctness of each implementation and library configuration with timing simulation by *Synopsys VCS MX Compiler Simulator* at two frequencies: 10 MHz and 100 KHz. The switching activity of each gate of the circuit was collected, and the average reported power measurements in Tables 3, 4 are obtained with *Synopsys Power Compiler*.

We outline some of the essential lightweight metrics of this paper’s architecture in Table 3. To the best of our knowledge, this is the smallest implementation of the all-in-one AES, which according to STM 90nm measurements achieves about 38% reduction in area compared to the previous work [5]. We further report the smallest stand-alone AES-192 and AES-256 versions in Table 4. The circuit offers flexibility to designers who might favor higher levels of security in this pre-quantum era by increasing the key size, at a reduced area cost. For example, since it has the same key length, our stand-alone implementation of AES-256 can be used to directly replace SKINNY-128-256 in recently proposed authenticated encryption candidates such as Romulus and SKINNY-AEAD from NIST LWC [18, 19]. One should notice that (under the same library UMC 90nm) although AES-256 (2197 GE, 1920 clock cycles) is slightly larger than SKINNY-128-256 (1937 GE, 8448 clock cycles [4]), it clearly has a marginal gain when it comes to latency. If we compare our combined AES-128/192/256 circuit to the one produced by *bit-sliding* [4], we can see that our circuit occupies a 36% more of area in GE but provides encryption and decryption for AES-192 and AES-256 which doubles the key path and increases considerably the control logic. Additionally, the latency is reduced 17% for encryption and 41 % for decryption in AES-128.

**Table 5:** Tabulation of the cell counts using the Nangate 15 nm library for each of the circuits

Cells					
circuit	AES-128/192/256	AES-192 e	AES-192 e/d	AES-256 e	AES-256 e/d
INV	141	56	113	63	101
AND2	163	62	92	54	96
AND3	14	2	7	2	3
AND4	14	3	5	5	5
OR2	254	141	218	143	210
OR3	9	4	9	2	4
OR4	6	6	6	4	2
NAND2	153	59	90	47	91
NAND3	14	2	7	2	3
NAND4	14	3	5	5	5
NOR2	147	88	116	85	115
NOR3	8	4	9	2	3
NOR4	5	6	6	4	2
MUX2	1	0	0	0	0
XOR2	102	50	96	53	85
XNOR2	61	51	57	41	64
AOI21	50	14	25	15	34
OAI21	57	19	42	23	34
OAI22	30	12	20	16	29
FA	3	0	0	0	0
HA	4	0	0	0	0
DFFSNQ	408	336	344	400	408

Note that DFFSNQ stands for D flip-flop with asynchronous reset. FA/HA stands for full/half adder, respectively. AOI21 represents the and-or-inverse gate $\overline{ A \vee (B \wedge C)}$. Similarly, OAI21 is the or-and-invert gate $\overline{ A \wedge (B \vee C)}$ and OAI22 similarly represents the gate $\overline{ (A\vee B) \wedge (C \vee D)}$. GATEx represents the corresponding gate with x bits of input and a single bit output. Further information can be found in [20]

In Table 4, we provide multiple results for different stand-alone versions of our circuit: AES-192 and AES-256, for encryption and decryption. To the best of our knowledge, each stand-alone version presented provides the first aim to produce a serial way circuit for this version, focusing on area minimization. To further give an idea of the circuit, in Table 5, we tabulate the number of standard cells that each circuit comprises of.

**Fig. 12:**
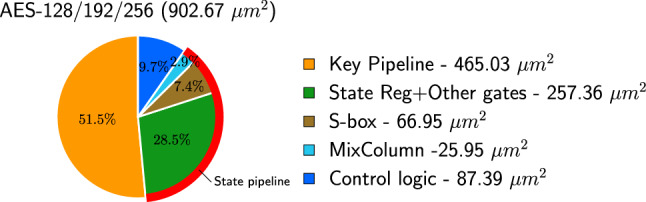
Breakdown of area occupied by individual components constructed using the Nangate 15nm cell library (using simple compiler directive)

In Fig 12, we give a breakdown of the area occupied by individual components in the circuit when constructed with Nangate 15 nm standard cells. Note that the area reported in the figure is 902.67 $\mu m^2$, whereas Table 3 reports the area of the same circuit as 753.99 $\mu m^2$. Note that the circuit reported in the table was constructed using the compile_ultra directive. Using this directive, the circuit synthesizer performs an additional optimization step to reduce the circuit area, but in the process it does not respect the boundaries between the individual components of the circuit. Hence, it is not possible to partition the final netlist so that each segment pertains to one particular module in the AES algorithm. However, if we do not direct the synthesizer to perform the additional optimization step, it returns a netlist that is sub-optimal in terms of area but “partitionable.” It is this circuit that is reported in Fig 12. In our experience, this also represents approximately the area occupied by the individual components in the optimal circuit obtained using compile_ultra.

### Power variations

Table 3 reports wide variations of power results between two clock frequencies for different cell libraries. To understand the reason for this, let us recap some basic facts about power consumption in CMOS transistors. There are 2 principal sources of power consumption in a CMOS circuit

1. Static: This is mainly caused due to the sub-threshold leakage current, which is the drain–source current in a CMOS gate when the transistor is OFF. This figure is becoming increasingly important as the technology is scaling down making the sub-threshold leakage more significant. Note that this component of the power consumption is independent of the frequency at which the input clock of the circuit is operated.
2. Dynamic: This is the power dissipated for charging and discharging the capacitive load of a gate when output transitions occur. This is essentially the total power consumed due to the combined effect of glitches and logic switching across all the nodes of the circuit. Note that this component is directly proportional to the clock frequency.

**Fig. 13:**
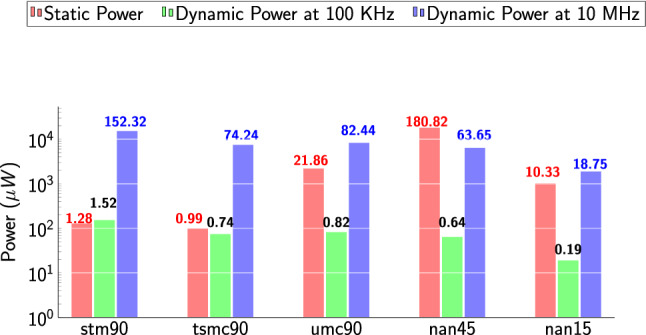
Breakdown of the static power and dynamic power consumed by the circuit at different frequencies in each of the cell libraries. Note that the y-axis is scaled logarithmically. All figures are in $\mu W$ units

We have synthesized our circuit with different cell libraries, each of which are constructed with CMOS transistors of varying feature sizes, which in turn consume varying amounts of static power. Figure 13 gives a breakup of different components of power over the two clock frequencies 100 KHz and 10 MHz. Note that the static power is same for both the frequencies for any given cell library. And for all cell libraries, the dynamic power consumed at 10 MHz is around 100 times the dynamic power consumed at 100 KHz, which follows from the fact that the dynamic component of power varies directly as the clock frequency. For example, using the STM 90nm library the circuit consumes 2.8 $\mu W$ at 100 KHz which is basically the sum of the static component $1.28~\mu W$ and the dynamic component $1.52~\mu W$ at 100 KHz. However, at 10 MHz the total power consumed is $153.6~\mu W= 1.28~\mu W \text {(static)} + 152.32~\mu W \text {(the dynamic component at 10 MHz)}$. All the power variations in the table can be decomposed as per the figures in Fig 13. In addition, there are also large differences in maximum throughput. This is mainly due to the fact that circuits constructed using CMOS transistors of lower feature length have naturally much lower signal delay across source to drain, and so the total critical path for these circuits is much less. Thus, circuits with smaller feature size cells like Nangate 15/45 nm can be operated at much higher clock frequency and hence have larger throughput.

### Comparison with FPGA implementations

Designing for FPGAs is indeed vastly different from designing for ASICs. On an ASIC platform, for the purpose of mapping a given logic into silicon, a circuit synthesizer usually has the liberty of choosing the best possible combination from a variety of standard cells already present in a library. Furthermore, this choice may change given the type of optimization required. For example, the area-optimized circuit of a given algorithm may vastly differ from its latency-optimized circuit, etc. However, this is not the case for FPGAs. Each FPGA device is composed of a finite number of logic elements called slices, each of which contain a predefined selection of gates. The challenge for designing for FPGAs is to make effective use of the resources offered by each slice.

There have been several papers that have attempted to reduce the size of AES on FPGAs [21–24]. The paper [25] contains a very nice introductory tutorial of how to optimize the AES circuit for the Spartan 6 FPGA family. FPGAs are reconfigurable hardware devices consisting of configurable logic blocks (CLB). In modern Xilinx FPGAs, each CLB is further subdivided into two slices that each contains four lookup tables (LUTs), eight registers and additional carry logic [26]. Each LUT can be used either to design one 6 variable Boolean function or two $5\times 1$ Boolean functions provided they are defined on the same input variables.

It is easy to see that an optimal FPGA implementation of the AES S-box requires 32 LUTs in eight slices, as each of its eight coordinate functions is an 8-to-1 mapping. Each 8-bit Boolean function requires four 6-to-1 LUTs to construct, and hence, the result follows. It was pointed out in [25] that there was no obvious way to reduce this number, as every linear combination of coordinate functions maintains the maximal algebraic degree of seven and depends on all eight input bits. Most of the area-optimized AES S-boxes in ASIC that are reported in the literature (i.e., the S-box of Maximov/Ekdahl that we use or the Canright S-box [27]) are not suited for FPGA implementation on Spartan 6 devices as they use tower field decomposition of $GF(2^8)$. As a result, they perform a lot of operations on *GF*(2)/*GF*(4) that lead to under-utilization of the 6-to-1 LUTs.

In [25], the authors found an ingenious way to fit the S-box in less number of slices. It is well known that the if the input byte is interpreted as an element of $GF(2^8)$ represented in the polynomial basis $x^8+x^4+x^3+x+1$, then the AES S-box can be expressed as the inverse function $x^{254}$ followed by an affine mapping. However, if the input is represented in any normal basis $\beta ,\beta ^2,\beta ^{2^2},\ldots ,\beta ^{2^7}$, then each coordinate of $x^{254}$ can be computed by applying the same function $S^*$ over bit-rotated versions of the input. So to compute the S-box, we would need the following:

a: An implementation of the 8-bit Boolean function $S^*$, which requires four 6-to-1 LUTs and therefore one slice.
b: A logic circuit p2n to change the input from the polynomial to a suitable normal basis. This is an 8-to-8 linear function.
c: A logic circuit n2p to change the output from the normal to the polynomial basis and compute the subsequent affine map. This is another 8-to-8 linear function.
d: A rotating register R1 that rotates the output of p2n that is input to $S^*$ to compute each coordinate of the inverse function, and another rotating register R2 that stores this output.

The authors searched $GF(2^8)$ exhaustively for a suitable element $\beta$ to construct the normal basis so that the logic for p2n, n2p, R1 and R2 can fit in 3 slices, which makes the total S-box fit in 4 slices. Although this is one half of a straightforward 8-slice implementation based on a lookup table, note that this construction actually takes 8 cycles to compute the S-box as opposed to the single cycle taken by the 8-slice implementation. We implemented our AES circuit on the same Spartan 6 device 6slx4, and we present the synthesis reports in Table 6. Note that although our implementation is better in terms of latency, in terms of area, area-wise, it is nowhere close to the circuit presented in [25] that is specifically optimized for this FPGA device.

**Table 6:** The synthesis reports for the Xilinx Spartan device 6slx4tqg144-2

Design	# LUTS	# FFs	# Slices	# Cycles	$f_{max}^\dagger$
Circuit in [25]	68	39	17	5538	109 MHz
AES-128/192/256	475	137	161	1408/1664/1920	51.2 MHz
AES-192 e	244	76	85	1664	64.3 MHz
AES-192 e/d	374	118	128	1664	53.9 MHz
AES-256 e	220	77	72	1920	76.8 MHz
AES-256 e/d	374	127	124	1920	59.7 MHz

$^\dagger :$ Note that $f_{max}$ is generated from the post-PAR simulation

### Protected implementation

We further introduce some ideas of how to adapt our circuit for a protected implementation of AES. Since a full description of the protected circuit is out of scope, let us introduce a small discussion as to how one could implement such a circuit. The only protected bit-serial S-box for AES was proposed in [25], and given that area size is one of the optimization goals, we feel that this architecture is best suited to be implemented with the data and key pipeline we have proposed. Note that the protected S-box in [25] only implements the forward S-box, but the inverse S-box can also be implemented with some minor modifications.

The formula for computing the S-box can be written as $\mathsf {Affine}(x^{254})$.^4^ The inverse S-box operation can therefore be written as $(\mathsf {Affine}^{-1}(x))^{254}$. Since the core nonlinear operation is still the inverse function $x^{254}$ over $GF(2^8)$, the inverse S-box is thus obtained by computing the $\mathsf {Affine}^{-1}$ operation just before the protected $x^{254}$ function.

In [25], the authors give a step-by-step description of how a threshold implementation (TI) of the bit-serialized S-box can be implemented. Although it was implemented on a FPGA device, the same principle can be applied on ASIC circuits. For the purpose of this discussion, we mention the salient points of their construction here. Note that in the previous subsection, we had mentioned that if the input is represented in any normal basis, then each coordinate of $x^{254}$ can be computed by applying the same function $S^*$ over bit-rotated versions of the input. Now, the same is true for any power function mapping over $GF(2^8)$. The algebraic degree of $S^*$ is 7, and since it is more difficult to construct TI of higher degree functions, the authors decompose the original power map into two cubic power maps $x^{254}= (x^{26})^{49}$. Since $x^{26}$ and $x^{49}$ are both power maps, they can be computed by repeated application of some Boolean function $F^*$ (resp. $G^*$) over rotated versions of the input bits when presented in a suitable normal basis. Moreover, since the Hamming weight of both 26 and 49 is 3, from elementary theory of power mappings we know that the algebraic degrees of both $F^*,G^*$ are 3, and so it is much easier to construct protected circuits for these Boolean functions.

The functions $F^*,G^*$ are further decomposed into functions $F^*=F^A+F^B$ and $G^*=G^A+G^B$, so that each can be shared using the (3, 1)-matrix sharing method [25, Eqn 1,2,4]. Each of $F^A,F^B,G^A,G^B$ can be shared using 2 input and 8 output shares for first-order security. So each $F^*, G^*$ is implemented using 2 input and 16 output shares. These 16 shares are then sent to a register bank where they are compressed back to 2 shares by XORing individual shares in the next clock cycle.

Therefore, the entire circuit will have the following components:

- An initial affine function: for the forward S-box, this is simply the function that converts from polynomial to normal basis. For the inverse S-box, it is the combination of the $\mathsf {Affine}^{-1}$ function and the polynomial to normal map.
- A shared implementation of $F^*$: this needs two rotating 8-bit registers R1A, R1B to rotate the 2 input shares, the shared circuit for $F^*$, another bank of 16 registers to store the output shares, a compression layer to XOR the 16 intermediate shares back to 2 shares and another two rotating registers S1A, S1B to store the output shares after compression.
- A shared implementation of $G^*$: this needs two rotating 8-bit registers R2A, R2B to rotate the 2 input shares, the shared circuit for $G^*$, another bank of 16 registers to store the output shares, a compression layer to XOR the 16 intermediate shares back to 2 shares and another two rotating registers S2A, S2B to store the output shares after compression.
- An final affine function: for the forward S-box, this is the function that converts from normal to polynomial basis combined with $\mathsf {Affine}$. For the inverse S-box, it is simply the function and the normal to polynomial map.

In the original paper, it took 26 cycles to compute one S-box function on the Spartan 6 device, and we think on ASIC platforms we could possibly do the same using some optimization. For example, R1A and R2A can be chosen to be some register $\mathsf {FF}_{x:x+7}$ and $\mathsf {FF}_{x+8:x+15}$ in the data pipeline, with some extra logic to insure that it can perform the circular internal rotation function. We would need additional registers for R1B, R2B. Similarly, R2A and R2B can simply be used as the output registers S1A and S1B of the $F^*$ layer. It takes 8 cycles for each byte to be shifted into R1A/B. Each output bit of $F^*,G^*$ takes 2 cycles to generate due to the additional compression layer. Hence, by effective pipelining the output bits of $F^*$ can be generated in cycles 9 to 17 and the same for $G^*$ is cycles 18-26. The initial and final affine layers can be done on the fly in cycles 9 and 26, making the entire S-box calculable in 26 cycles. To accommodate this into the entire data pipeline using minimum cycles is a challenging problem in the engineering sense and indeed subject of our future investigations.

## Footnotes

1 See Infineon jTOP ID SLJ 52GCA150CL Java Card 3.0.4 150K as an example.

2 Some of these D flip-flops are later upgraded to scan flip-flops, which has the capability of choosing between two inputs. We still refer to them by the variable FF.

3 Alternatively, this can also be done with a scan flip-flop.

4 Note that the $\mathsf {Affine}(x^{254})$ notation is a simplified one for the purposes of better understanding, and not strictly mathematically correct. This is because the $x^{254}$ is carried out over $GF(2^8)$, and the affine function is a matrix multiplication operation done by treating the output of the $x^{254}$ operation as an 8-bit vector over GF(2).
